# Blood viscosity is associated with smoking status rather than the presence of chronic obstructive pulmonary disease: findings from the Korea COPD Subgroup Study (KOCOSS)

**DOI:** 10.1186/s12931-026-03827-8

**Published:** 2026-08-04

**Authors:** Ye Jin Lee, Hye-Rin Kang, Sang Hyuk Kim, Hyun Jung Kim, Jae Seung Lee, Chang-Hoon Lee, Sung Kyoung Kim, Yu-Il Kim, Kwang Ha Yoo, Youlim Kim

**Affiliations:** 1 Division of Pulmonary and Critical Care Medicine, Department of Internal Medicine, Seoul National University Bundang Hospital, Seoul National University College of Medicine, Seongnam, Republic of Korea; 2 Division of Pulmonary and Allergy, Department of Internal Medicine, Hallym University Dongtan Sacred Heart Hospital, Hwaseong, Republic of Korea; 3 Division of Pulmonary, Allergy, and Critical Care Medicine, Department of Internal Medicine, Korea University Guro Hospital, Korea University College of Medicine, Seoul, Republic of Korea; 4 Division of Pulmonary and Critical Care Medicine, Department of Internal Medicine, Keimyung University Dongsan Hospital, Keimyung University School of Medicine, Daegu, Republic of Korea; 5 Division of Pulmonology and Critical Care Medicine, Department of Internal Medicine, Asan Medical Center, University of Ulsan College of Medicine, Seoul, Republic of Korea; 6 Division of Pulmonary and Critical Care Medicine, Department of Internal Medicine, Seoul National University Hospital, Seoul, Republic of Korea; 7 Division of Pulmonary and Critical Care Medicine, Department of Internal Medicine, College of Medicine, St. Vincent’s Hospital, The Catholic University of Korea, Seoul, Republic of Korea; 8 Division of Pulmonology, Department of Internal Medicine, Chonnam National University Hospital, Chonnam National University Medical School, Gwangju, Republic of Korea; 9 Division of Pulmonary, Allergy and Critical Care Medicine, Department of Internal Medicine, Konkuk University Medical Center, Konkuk University School of Medicine, Seoul, Republic of Korea

**Keywords:** Blood viscosity, Chronic obstructive pulmonary disease, Lung function decline, Smoking

## Abstract

**Background:**

The relationship between smoking and blood viscosity is well established. However, the role of blood viscosity in the development of chronic obstructive pulmonary disease (COPD) and its impact on lung function decline remains unclear. Therefore, we aimed to investigate the association of blood viscosity with COPD and lung function decline, independent of smoking status.

**Methods:**

Data from 292 participants in the Korea COPD Subgroup Study (KOCOSS), a multicenter prospective cohort, were analyzed. Subjects were classified into four groups based on smoking history and COPD status. Multiple linear regression analysis was performed to determine the association between baseline viscosity and lung function decline after adjusting for age, sex, smoking status, and COPD status.

**Results:**

Of the 292 participants, 93 (31.8%) patients had COPD. Ever smokers with or without COPD were older than never smokers and predominantly male. Never smokers with COPD reported the highest scores for the Modified Medical Research Council Dyspnea Scale. Blood viscosity was significantly higher in ever smokers (with COPD: 9.0 ± 1.4; without COPD 9.2 ± 1.5) than in never smokers (with COPD: 8.7 ± 1.8; without COPD: 8.4 ± 1.5). Viscosity did not differ significantly with the presence of COPD within the same smoking category. Adjusted multiple linear regression revealed that baseline blood viscosity was not associated with 2-year declines in forced expiratory volume in 1 s (*p* = 0.557), forced vital capacity (*p* = 0.686), or diffusing capacity of the lung for carbon monoxide (*p* = 0.947).

**Conclusion:**

Blood viscosity appeared to be more closely associated with smoking-related hemorheological changes than with COPD. Its potential as a predictive biomarker for lung function decline appears to be limited, necessitating further long-term investigation.

**Supplementary Information:**

The online version contains supplementary material available at 10.1186/s12931-026-03827-8.

## Background

Persistent low or rapidly declining lung function trajectories are not only observed in patients with chronic obstructive pulmonary disease (COPD) but also in healthy adults with chronic or intermittent productive cough [1]. Interest in identifying the specific trajectory that leads to COPD and the factors associated with worsening lung function trajectories is growing. This is attributable to the association of rapid decline in lung function with adverse outcomes, including increased number of exacerbations, deteriorating quality of life, and increased frequency of cardiovascular events [2, 3]. The number of exacerbations is a well-established factor associated with lung function decline in COPD, and this has already been proven in randomized controlled trials [4–6]. Age, smoking status, existence and extent of emphysema, socioeconomic status, body mass index (BMI), and degree of dyspnea have also been identified as risk factors that contribute to the accelerated decline of lung function [7, 8]. Although several blood-based biomarkers for COPD have been investigated, their clinical utility differs substantially. Peripheral blood eosinophil count is currently the most established blood-based biomarker for COPD, serving as a surrogate marker associated with exacerbation risk, lung function decline, and response to inhaled corticosteroids or type 2 inflammation–targeted therapies [9, 10]. In contrast, other blood-based markers such as C-reactive protein (CRP), fibrinogen, or interleukin-6 lack sufficient specificity, consistency, or clinical relevance and are not routinely recommended for individual risk stratification or treatment decision-making [11–14].

Recent studies have effectively established associations of blood biomarkers with treatable traits in subjects with risk factors in early phase studies involving large prospective cohorts, including COPDGene and SPIROMICS [15]. Smoking is a significant risk factor for COPD. Approximately 20% of smokers develop the disease [16], but a substantial proportion of COPD cases occurs in never smokers, suggesting factors beyond smoking exposure contribute to the disease’s pathogenesis [17]. Although the genes analyzed vary from study to study, several studies have shown that genetic variation in individuals influences differential susceptibility to COPD [18]. However, reliable blood-based markers that can predict COPD development or rapid lung function decline in both smokers and never smokers remain limited. Cigarette smoke exposure induces oxidative stress, which plays a crucial role in the pathogenesis of COPD [19, 20]. The imbalance between oxidants and antioxidants triggered by smoking leads to chronic lung inflammation and contributes to COPD development and progression [21]. Oxidative stress has also been linked to red blood cell (RBC) aggregation associated with increased blood viscosity [22–24].

Previous studies have shown that increased blood viscosity impairs pulmonary blood flow in the general population [25]. However, the specific role of blood viscosity in COPD—particularly in differentiating the effects of the disease from those of smoking—remains poorly characterized. Furthermore, the impact of viscosity on longitudinal lung function decline has not been explored in smoking or non-smoking COPD populations. Therefore, we aimed to evaluate whether blood viscosity was independently associated with COPD and lung function decline independently of the effects of smoking exposure.

## Methods

### Study population and study design

This study involved a secondary analysis of data from the Korea COPD Subgroup Study (KOCOSS), a multicenter prospective longitudinal cohort study designed to follow patients across 55 nationwide referral hospitals using a standardized protocol. For the current analysis, we used data collected between 2021 and 2023. During this period, the study protocol was expanded to include blood viscosity measurements of the enrolled participants and the data were prospectively collected. The cohort was recruited to cover a wide spectrum of the disease and included patients with confirmed COPD, those with non-obstructive but at-risk respiratory phenotypes such as preserved ratio-impaired spirometry and chronic bronchitis, ever smokers without airflow obstruction, and non-smoker healthy controls. The KOCOSS cohort comprised participants in a stable state, which was defined as having no acute exacerbations or respiratory infections requiring medication changes for at least 4 weeks prior to enrollment. In the present study, we included participants aged ≥ 40 years from the KOCOSS cohort, including healthy controls and individuals with and without COPD. Eligible participants were required to have available baseline data on blood viscosity, spirometry, and smoking history. COPD was diagnosed based on the ratio of post-bronchodilator forced expiratory volume in 1 s (FEV_1_) to forced vital capacity (FVC) (FEV_1_/FVC) of < 0.70. Participants were excluded if they had missing baseline FEV_1_/FVC values (*n* = 6) or smoking status (*n* = 2). After applying these criteria, 292 participants from the initial 300 enrolled in the KOCOSS cohort were included.

The participants were scheduled to undergo annual follow-up pulmonary function tests for 2 years. Blood viscosity was measured at baseline during 2021–2023 to investigate its association with smoking and COPD statuses. For the baseline cross-sectional analysis, all 292 participants were included. For the longitudinal complete-case analysis of lung function decline, participants were required to have baseline, 1-year, and 2-year FEV_1_, FVC, and diffusing capacity of the lung for carbon monoxide (DLCO) data. In total, 102 participants met these criteria and were included for the longitudinal analysis. The remaining 190 participants were classified as dropouts during the attrition analysis, and their data were excluded from the longitudinal regression models. The following demographic information of the participants were collected: age, sex, address, level of education, smoking history, past medical history including respiratory-specific disease, and BMI. For comparative analysis, the participants were categorized into four groups based on their smoking history and COPD status: (1) never smokers without COPD, (2) never smokers with COPD, (3) ever smokers without COPD, and (4) ever smokers with COPD. This stratification allowed for the evaluation of the association between blood viscosity and COPD presence, independent of the effects of smoking exposure.

### Clinical assessment and data collection

Respiratory symptoms and quality of life were evaluated using standardized questionnaires, including the modified Medical Research Council (mMRC) Dyspnea Scale, the COPD Assessment Test, and the COPD-specific version of the St. George’s Respiratory Questionnaire. The comorbidity burden was assessed using the Charlson Comorbidity Index (CCI) and based on the documented medical history of the participants. Clinical parameters such as blood pressure, complete blood count, hematocrit, blood urea nitrogen, creatinine, and liver function were measured for each participant. In addition to these clinical data, we collected blood samples, induced sputum, and pulmonary function with lung volume results for these groups. The participants were scheduled to undergo annual follow-up lung function tests. While the longitudinal analysis focused on complete data collected at baseline, 1 year, and 2 years, participants without follow-up data were retained in the study for baseline cross-sectional comparisons.

### Measurement of blood viscosity as a biomarker

The blood samples from the KOCOSS cohort were collected in tubes containing ethylenediaminetetraacetic acid as anticoagulant and sent directly to the Green Cross Reference Laboratory for standardized viscosity analysis. Before analysis, samples were stored under refrigerated conditions at 2–8 °C and measured within 2 days of sample collection according to the laboratory protocol. Whole-blood viscosity was measured using the cone-plate rotational method with a ZL-6000P analyzer (Zonci Technology). Measurements were performed at 37 °C, which corresponded to the physiological body temperature. Whole-blood viscosity was measured under two shear-rate conditions and reported as systolic and diastolic blood viscosities. Systolic blood viscosity corresponded to high-shear viscosity measured at a shear rate of 300 s⁻¹, whereas diastolic blood viscosity corresponded to low-shear viscosity measured at a shear rate of 5 s⁻¹. In the present study, diastolic blood viscosity was selected as the primary variable because it is more sensitive to red blood cell aggregation and microcirculatory flow resistance [26], which are considered biologically relevant to smoking-related hemorheological changes and chronic lung diseases [27]. Briefly, a defined volume of whole blood was placed between the cone and plate, and viscosity was calculated from the resistance generated during cone rotation. Viscosity values were reported in mPa·s. The laboratory-provided reference ranges were 3.5–4.1 mPa·s for men and 3.0–3.6 mPa·s for women for systolic blood viscosity and 9.4–13.0 mPa·s for men and 7.6–11.1 mPa·s for women for diastolic blood viscosity. All measurements were performed according to the standardized operating procedures of the Green Cross Reference Laboratory, and the analyzer was regularly calibrated and subjected to quality control procedures to ensure reliability and reproducibility of the measurements [28, 29].

### Ethical considerations

The study was conducted in accordance with the principles of the Declaration of Helsinki. The study protocol was approved by the Institutional Review Board of Konkuk University Hospital (approval number: KUH1010338), which served as the main participating center and the Ethics Committee of each participating medical center. All participants provided written informed consent before enrollment, and all data were de-identified to ensure anonymity.

### Statistical analysis

Associations among blood viscosity, smoking history, COPD status, baseline demographics, laboratory findings, and lung function parameters for the four stratified groups were compared. The normality of the data was assessed using the Shapiro-Wilk test. Student’s t-test and one-way analysis of variance followed by the Games-Howell post hoc test were performed to evaluate the differences in continuous variables among different groups of patients. Continuous variables are presented as means ± standard deviation or median with interquartile ranges (IQR). For non-normally distributed variables, the Kruskal-Wallis test followed by the Dunn-Bonferroni post-hoc test was performed. Categorical variables were compared using the chi-squared or Fisher’s exact test and are summarized as frequencies with percentages (%).

Multiple linear regression was performed to identify the independent associations of baseline blood viscosity with the 2-year longitudinal changes in FEV_1_, FVC, and DLCO. Longitudinal changes in FEV_1_ and FVC were calculated as the difference between the baseline and 2-year follow-up post-bronchodilator spirometric values, which is consistent with the definition of COPD. Changes in the DLCO were calculated using standard DLCO measurements. To prevent simultaneously entering smoking status and COPD status as separate covariates, participants were modeled using a single combined four-level smoking/COPD group variable: never smokers without COPD, never smokers with COPD, ever smokers without COPD, and ever smokers with COPD. Never smokers without COPD were selected as the reference group because they represented the least clinically exposed. Only 102 participants had complete longitudinal pulmonary function data, which were the baseline, 1-year, and 2-year measurements of FEV_1_, FVC, and DLCO. Therefore, the primary model was intentionally parsimonious to reduce the risk of overfitting. The primary model included age, sex, four-level smoking/COPD group, and baseline blood viscosity. Covariates were selected based on clinical relevance, prior evidence, and data availability rather than automated selection based solely on univariate statistical significance. No automated variable selection method, such as stepwise selection or penalized regression, was used because of the limited longitudinal sample size and explanatory purpose of the analysis. Additional extended models were developed by sequentially adding BMI, inhaled medication use, and inflammatory markers (including CRP and white blood cell [WBC] count) to assess the robustness of the association between blood viscosity and changes in lung function. Multicollinearity was assessed using variance inflation factors, and model assumptions were evaluated using residual diagnostics. Based on previous research indicating that hematocrit is a major determinant of whole-blood viscosity [23], we conducted an a priori pairwise correlation analysis of hematocrit and blood viscosity. Given the strong correlation between these variables, hematocrit was not included in the primary multivariate model to prevent potential over-adjustment by controlling for a major biological component of the exposure itself. Pairwise correlations between pack-years and blood viscosity or hematocrit were also assessed via exploratory analyses.

To assess potential selection bias due to loss to follow-up, we performed an attrition analysis comparing the baseline characteristics of the participants with and without 2-year follow-up pulmonary function data. All statistical analyses were performed using SAS software (version 9.4; SAS Institute, Cary, NC, USA).

## Results

### Baseline characteristics of the study population

**Fig. 1:**
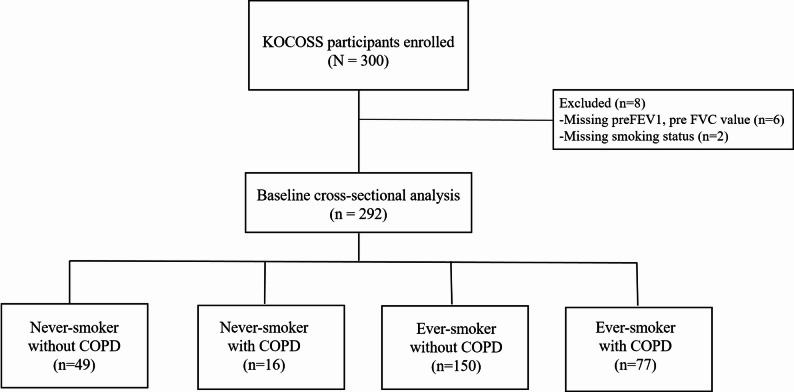
Flow chart of study population

Of 292 participants who had blood viscosity data, 93 (31.8%) were satisfied with the requirements for COPD diagnosis. Based on smoking history and the presence of COPD, we classified the participants into four groups: (1) never smokers without COPD (*n* = 49), (2) never smokers with COPD (*n* = 16), (3) ever smokers without COPD (*n* = 150), and (4) ever smokers with COPD (*n* = 77) (Fig. 1). The demographic characteristics of the participants stratified by smoking history and the presence or absence of COPD are shown in Table 1. Ever smokers with COPD were older than never smokers with COPD and were predominantly male. Participants without COPD tended to have lower COPD Assessment Test and COPD-specific version of the St. George’s Respiratory Questionnaire symptom scores than those with COPD.

**Table 1:** Baseline characteristics of study participants

Variables	*N*	Never smoker without COPD	Never smoker COPD	Ever smoker without COPD	Ever smoker COPD	*P*-value
		(*N* = 49)	(*N* = 16)	(*N* = 150)	(*N* = 77)	
Sex (Male, n (%))	292	16(32.65)	9(56.25)	143(95.33)	75(97.4)	< 0.001
Age, years	292	59.9 ± 13.79	49.75 ± 22.04	61.54 ± 11.22	64.32 ± 13.28	0.049
≤ 40 yr		5(10.2)	7(43.75)	5(3.33)	4(5.19)	< 0.001
40–49 yr		7(14.29)	2(12.5)	19(12.67)	10(12.99)	
50–59 yr		8(16.33)	0(0)	31(20.67)	10(12.99)	
60–69 yr		18(36.73)	4(25)	67(44.67)	20(25.97)	
70–79 yr		7(14.29)	2(12.5)	24(16)	28(36.36)	
≥ 80yr		4(8.16)	1(6.25)	4(2.67)	5(6.49)	
Education						0.108
≤ Middle school		17(34.69)	4(25)	36(25.71)	13(17.11)	
High school		11(22.45)	5(31.25)	56(40)	37(48.68)	
≥ College		21(42.86)	7(43.75)	48(34.29)	26(34.21)	
Smoking amount (pack-year)				33.5 ± 24.75	31.21 ± 33.74	0.585
BMI, kg/m^2^	292	23.85 ± 3.19	22.78 ± 2.83	24.47 ± 3.53	24.14 ± 3.36	0.236
< 18.5 (underweight)		0(0)	1(6.25)	7(4.67)	4(5.19)	0.354
18.5–22.9 (normal)		21(42.86)	7(43.75)	38(25.33)	23(29.87)	
23.0-24.9 (overweight)		13(26.53)	4(25)	39(26)	21(27.27)	
≥ 25(obese)		15(30.61)	4(25)	66(44)	29(37.66)	
SBP (mmHg)	234	125.34 ± 16.24	120.25 ± 14.14	129.55 ± 31.51	126.42 ± 14.77	0.536
DBP (mmHg)	234	75.54 ± 9.29	75.58 ± 8.68	77.9 ± 12.16	74.04 ± 12.52	0.203
Heart rate (/min)	230	75.61 ± 12.27	78.75 ± 12.49	79.29 ± 13.23	80.33 ± 12.65	0.335
mMRC grade	289	0.65 ± 0.86	1.31 ± 0.79	0.63 ± 0.73	0.91 ± 0.84	0.002
< 2		43(87.76)	10(62.5)	134(90.54)	63(82.89)	0.012
≥ 2		6(12.24)	6(37.5)	14(9.46)	13(17.11)	
CAT score	289	7.96 ± 7.44	10.88 ± 6.55	8.45 ± 6.42	10.03 ± 6.86	0.164
< 10		35(71.43)	8(50)	95(64.19)	42(55.26)	0.204
≥ 10		14(28.57)	8(50)	53(35.81)	34(44.74)	
SGRQ-c						
Symptom	288	23.8 ± 17.81	29.24 ± 13.15	22.09 ± 17.35	27.97 ± 23.22	0.116
Activity	288	15.74 ± 18.45	18.81 ± 15	15.33 ± 17.8	21.54 ± 20.16	0.106
Impact	288	9.90 ± 14.57	7.70 ± 8.66	8.83 ± 12.65	10.20 ± 14.32	0.834
Total	288	14.14 ± 13.98	14.90 ± 8.94	13.16 ± 12.72	16.82 ± 15.33	0.296
WBC count (10^3^/mm^3^)	291	5.48 ± 1.54	6.34 ± 1.04	6.55 ± 2.16	6.59 ± 2.22	0.011
Hemoglobin (g/dL)	291	13.03 ± 1.19	13.66 ± 1.73	14.4 ± 1.50	14.39 ± 1.41	< 0.0001
Hematocrit (%)	291	39.32 ± 3.74	40.54 ± 5.07	42.74 ± 4.24	42.94 ± 4.36	< 0.0001
Uric acid (mg/dL)	290	4.55 ± 1.2	5.11 ± 1.35	5.46 ± 1.37	5.77 ± 1.44	< 0.0001
AST (IU/L)	291	22.71 ± 6.19	23.44 ± 6.94	24.45 ± 7.97	30.67 ± 38.33	0.106
ALT (IU/L)	291	19.04 ± 8.77	18.31 ± 8.11	23.64 ± 13.27	27.75 ± 22.24 §	0.009
Total bilirubin (mg/dL)	291	0.35 ± 0.17	0.51 ± 0.2	0.49 ± 0.26	0.49 ± 0.27	0.005
Creatinine (mg/dL)	291	0.81 ± 0.16	0.87 ± 0.27	0.98 ± 0.21	1.11 ± 0.77	0.002
BUN (mg/dL)	291	14.92 ± 4.26	15.69 ± 4.7	16.38 ± 4.47	17.68 ± 7.06	0.035

Values are expressed as mean ± standard deviation (SD) for the continuous variables, and frequencies with percentage (%) for categorical variables

*Abbreviations:* *AST* Aspartate aminotransferase, *ALT* Alanine aminotransferase, *BMI* Body mass index, *BUN* Blood urea nitrogen, *CAT *score, COPD Assessment Test, *DBP* Diastolic blood pressure, *mMRC* modified Medical Research Council Dyspnea Scale, *SBP* systolic blood pressure, *SGRQ-C* COPD-specific version of St. George’s Respiratory Questionnaire, *WBC* White blood cell count

Never smokers with COPD reported the highest mMRC dyspnea scores (1.31 ± 0.79, *p* = 0.002), which exceeded the mean scores of the other three groups, which remained below 1. Further, ever smokers (regardless of COPD status) had significantly higher WBC count, hematocrit, and uric acid, alanine aminotransferase, creatinine, and blood urea nitrogen concentrations than never smokers without COPD. These findings suggest that these laboratory abnormalities are linked to smoking exposure rather than the pathophysiology of COPD (Table 1). Pairwise comparisons of the baseline characteristics are provided in Supplementary Table S1. In these comparisons, blood viscosity was higher in ever smokers with or without COPD than in never smokers without COPD but did not differ significantly with COPD status within the same smoking category.

### Differences in blood viscosity based on the presence of COPD and smoking history

**Fig. 2:**
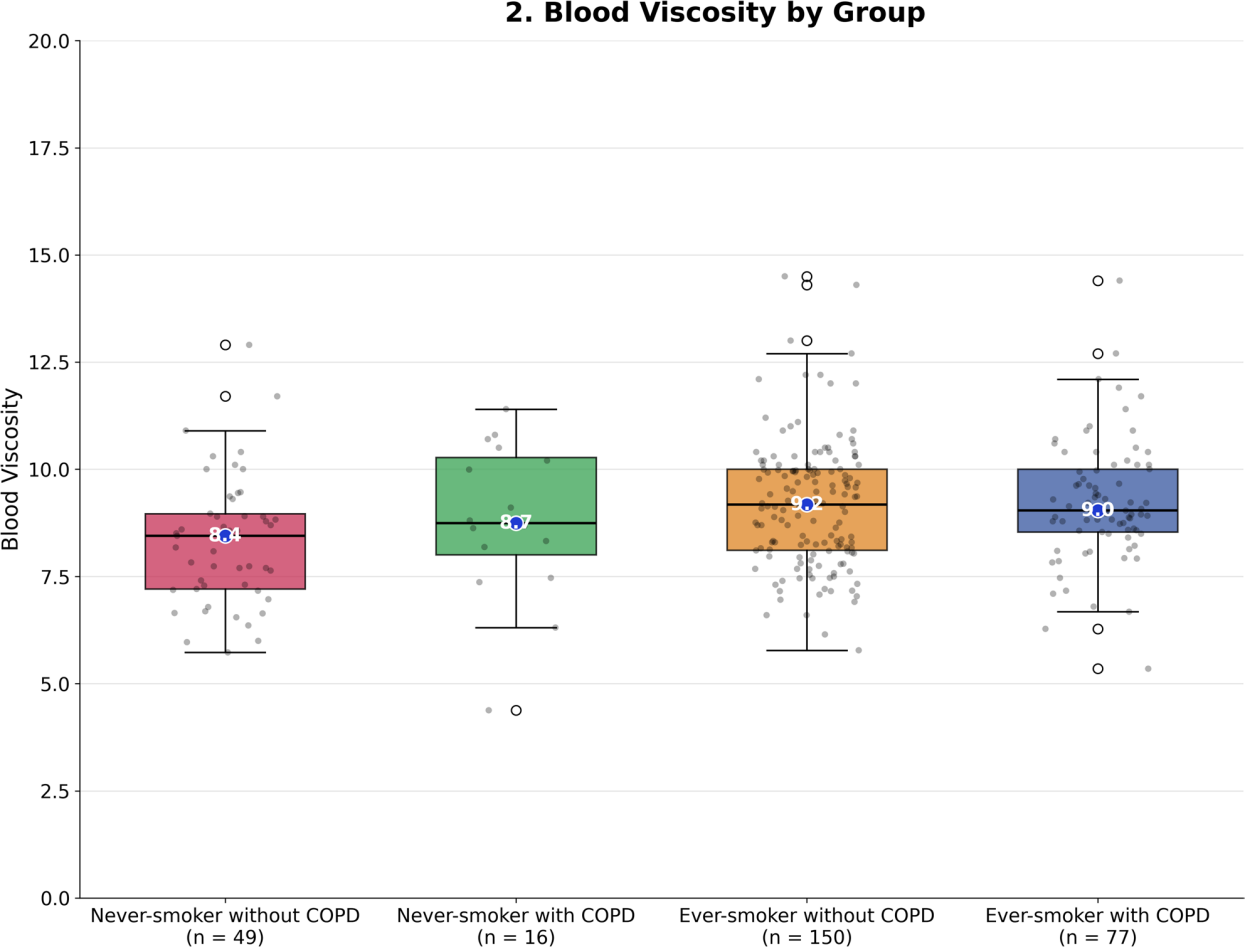
Comparison of blood viscosity difference by smoking and COPD status

Blood viscosity was significantly higher in ever smokers with COPD (9.0 ± 1.4) and without COPD (9.2 ± 1.5) than never smokers without COPD (8.4 ± 1.5; *p =* 0.002 for both comparisons). However, there were no significant differences in blood viscosity between individuals with and without COPD within the same smoking status group (never smokers, *p =* 0.840; ever smokers, *p =* 0.919). Taken together, these results suggest that blood viscosity is more consistently related to smoking status than to COPD, although subgroup-specific comparisons should be interpreted cautiously because of the small number of never smokers with COPD (Fig. 2).

### Baseline lung function and impact of viscosity on lung function trajectories

Of the 292 participants, 102 had complete baseline, 1-year, and 2-year FEV_1_, FVC, and DLCO data, and were included in the longitudinal complete-case analysis. An attrition analysis comparing the completers and dropouts is presented in Supplementary Table S2. The completers were older, more likely to be ever smokers, and had different baseline pulmonary function indices, suggesting that attrition was not random. However, the baseline blood viscosity, BMI, hematocrit, hs-CRP, CCI, COPD status, and four-level smoking/COPD group distribution of the groups were comparable. Thus, loss to follow-up was not clearly related to baseline blood viscosity or the main smoking/COPD classification, although selection bias related to age, smoking status, and baseline lung function could not be excluded. Ever smokers, regardless of the COPD status, had a higher FVC than never smokers (*p* < 0.001). Although FVC % predicted was the lowest for the never-smokers with COPD group (80% [IQR 68–97]), no significant differences were observed across the four groups (*p =* 0.466). Never smokers with COPD had the lowest FEV_1_ (1.66 L, [IQR 1.16–2.68]) relative to those of ever smokers with (2.23 L, [IQR 1.61–2.76]) and without (2.78 L, [IQR 2.45–3.26]) COPD, and never smokers without COPD (2.31 L, [IQR 1.86–2.83]) (*p* < 0.001). This pattern of significant impairment in never smokers with COPD was consistently observed in these parameters of the % predicted FEV_1_, FEF_25−75%_, FEV_1_/FVC ratio (%), and DLCO (Table 2). The results of the pairwise comparisons of the baseline lung function parameters are provided in Supplementary Table S3, and they support the greater airflow limitation observed in the COPD group, particularly FEV_1_% predicted, FEF25–75% % predicted, and FEV_1_/FVC.

**Table 2:** Baseline lung function test of study participants

Variables	*N*	Never smoker without COPD	Never smoker COPD (*N* = 16)	Ever smoker without COPD (*N* = 150)	Ever smoker COPD	*p*-value
		(*N* = 49)			(*N* = 77)	
FVC, L	292	3(2.49, 3.52)	3.03(2.54, 3.76)	3.84(3.33, 4.43)	3.94(3.29, 4.37)	< 0.001
FVC, % pred	292	88(78, 94)	80(68, 97)	87(79, 98)	87(82, 98)	0.466
FEV_1_, L	292	2.31(1.86, 2.83)	1.66(1.16, 2.68)	2.78(2.45, 3.26)	2.23(1.61, 2.76)	< 0.001
FEV_1_, % pred	292	87(76, 97)	61.5(40.5, 84)	86(78, 96)	73(57, 85)	< 0.001
FEF _25−75%_, % pred	292	80(54, 96)	21.5(16, 66.5)	68.5(56, 95)	33(19, 47)	< 0.001
FEV_1_/FVC, %	292	77(73, 80)	54.5(49, 73)	73(70, 78)	58(51, 65)	< 0.001
BD* FVC, L	285	3.03(2.51, 3.57)	3.1(2.5, 3.88)	3.78(3.31, 4.34)	3.96(3.3, 4.39)	< 0.001
BD FVC, % pred	285	87(79, 96)	79(65, 98)	86(78, 97)	88.5(81, 98.5)	0.264
BD FEV_1_, L	285	2.38(1.94, 3.03)	1.91(1.18, 3.22)	2.85(2.54, 3.33)	2.25(1.72, 2.9)	< 0.001
BD FEV_1_, % pred	285	91(79, 101)	50(45, 85)	88(80, 98)	78(58, 88)	< 0.001
BD FEF_25−75%_, % pred	285	87(65, 105)	21(18, 87)	78(64, 97)	39.5(23, 49.5)	< 0.001
BD FEV_1_/FVC, %	285	79(74, 82)	57(52, 80)	75(71, 79)	62(54, 66.5)	< 0.001
DLCO, mL/mmHg/min	278	16(13.6, 18.6)	15.1(9.5, 20.6)	18.2(14.4, 22.4)	16.5(12.8, 19.3)	0.02
DLCO, % pred	278	88(80, 96)	76(63, 94)	85(70, 96)	77(61, 90)	0.001
DLVA, mL/mmHg/min	278	4.21(3.68, 4.58)	4.15(3.07, 4.85)	3.76(3.25, 4.2)	3.38(2.39, 4.05)	< 0.001
DLVA, % pred	278	84(75, 91)	87(77, 103)	90(79, 99)	83(58, 99)	0.044

Values are expressed as medians and interquartile ranges for non-normally distributed variables

*Abbreviations: BD* Bronchodilator, *DLCO* Diffusing capacity of the lung for carbon monoxide, *DLVA* Alveolar Volume Adjusted DLCO, *FEF25-75% *Forced Expiratory flow, *FEV*_1_ Forced Expiratory Volume in 1 s, *FEV*_1_/FVC ratio of Forced Expiratory volume in 1 s to Forced Vital capacity, *FVC* Forced Vital Capacity, *% pred* percent predicted

Hematocrit showed a strong positive correlation with overall blood viscosity before the longitudinal analysis (Pearson *r* = 0.640, *p* < 0.001), and similar correlations were observed for the smoking subgroups. In the exploratory analyses of ever smokers, pack-years were not significantly correlated with blood viscosity in ever smokers overall or within the COPD-stratified ever-smoker subgroups (Supplementary Table S4). To evaluate the independent impact of blood viscosity on longitudinal lung function changes, we analyzed the 2-year changes in lung function after adjusting for age, sex, and combined four-level smoking/COPD group variables (Table 3). In the adjusted multiple linear regression analysis, baseline blood viscosity was not significantly associated with the 2-year decline in FEV_1_ (*p* = 0.557), FVC (*p* = 0.686), or DLCO (*p* = 0.947). This association remained consistent across extended models that sequentially incorporated BMI, inhaled medication use, CRP concentration, and WBC count. Male sex was consistently associated with a greater increase in FEV_1_% predicted, whereas the never-smoker COPD group showed a greater increase in FVC % predicted than the never-smoker non-COPD reference group (Supplementary Table S5). Multicollinearity diagnostics did not indicate substantial collinearity among the variables included in the primary models (Table 3). The maximum variance inflation factor was 2.77, and all VIF values were below 5: age, 1.09; male sex, 1.40; never smoker with COPD, 1.24; ever smoker with COPD, 2.64; ever smoker without COPD, 2.77; blood viscosity, 1.13. Residual diagnostics did not show major violations of the linear regression assumptions (Supplementary Table S6). Age and smoking status were not significantly associated with lung function trajectories in the final model.

**Table 3:** Association between blood viscosity and lung function change

Variable	FEV_1_% Predicted 2-Year Change		*P*	FVC % Predicted 2-Year Change	*P*	DLCO % Predicted 2-Year Change	*P*
Age	-0.08 (-0.25 to 0.09)	0.337		-0.13 (-0.27 to 0.02)	0.090	-0.26 (-0.55 to 0.02)	0.067
Sex, Male	7.59 (1.70 to 13.47)	0.012		4.07 (-1.10 to 9.25)	0.121	4.65 (-5.34 to 14.64)	0.357
Never smoker + non-COPD	ref			ref		ref	
Smoker + non-COPD	-0.09 (-5.46 to 5.28)	0.974		1.09 (-3.63 to 5.80)	0.648	-1.78 (-10.89 to 7.34)	0.699
Never smoker + COPD	11.05 (-0.80 to 22.90)	0.067		15.47 (5.06 to 25.88)	0.004	5.26 (-14.85 to 25.37)	0.604
Smoker + COPD	-0.31 (-6.02 to 5.41)	0.916		-0.11 (-5.13 to 4.91)	0.965	1.07 (-8.63 to 10.77)	0.827
Blood viscosity	0.34 (-0.80 to 1.48)	0.557		0.20 (-0.80 to 1.20)	0.686	-0.07 (-2.00 to 1.87)	0.947
Final model fit	Adj. R^2^: 0.063, AIC: 622.5			Adj. R^2^: 0.081, AIC: 599.5		Adj. R^2^: -0.015, AIC: 716.6	

Values are presented as beta coefficients (95% CI) for linear regression models

*Abbreviations: Adj. R² *Adjusted R-squared, *AIC* Akaike Information Criterion, *COPD* Chronic Obstructive Pulmonary Disease, *DLCO* Diffusing Capacity of the Lung for Carbon Monoxide, *FEV*_1_ Forced Expiratory Volume in 1 Second, *FVC* Forced Vital Capacity

## Discussion

To the best of our knowledge, this is the first study to investigate the association between blood viscosity and the presence of COPD and its impact on longitudinal lung function trajectories. There was no significant difference in blood viscosity between never smokers with and without COPD or between ever smokers with and without COPD. These findings suggest that COPD is not directly associated with systemic blood viscosity. Furthermore, blood viscosity was not a potential predictor of the 2-year longitudinal changes in FEV_1_, FVC, or DLCO. Our results indicate that blood viscosity may reflect smoking-related hemorheological changes rather than the presence of COPD, although its utility as a predictive biomarker of longitudinal decline in lung function appears to be limited.

The elevation in blood viscosity in ever smokers, independent of their COPD status, was consistent with the findings of previous studies [30–32] demonstrating that hemorheological alterations are the primary response to tobacco smoke. While some studies have reported an association between COPD and increased viscosity, such findings are typically limited to patients with acute exacerbations [24] or those with decompensated chronic cor pulmonale [33]. In contrast, the results of our study, which focused on stable patients, are consistent with prior evidence showing that blood viscosity does not significantly increase in patients with stable stage 1–2 COPD. Blood viscosity remained within normal range in never smokers with COPD despite their severe airflow obstruction (FEV_1_ 61.5%), and this reinforced that stable COPD pathophysiology does not necessarily trigger systemic hyperviscosity, regardless of severity. This suggests that the rheological alterations observed in smokers represent a specific response to tobacco exposure rather than a fundamental feature of COPD.

Our hematological observations further support this interpretation. Hematocrit is a well-known factor that influences blood viscosity [32]. In our study, it was significantly higher in ever smokers than in never smokers, regardless of the COPD status (*p* < 0.001). However, it did not differ significantly with the presence of COPD within the same smoking category (never smokers: 40.54 ± 5.07 vs. 39.32 ± 3.74, *p* = 0.320; ever smokers: 42.94 ± 4.36 vs. 42.74 ± 4.24, *p* = 0.990). Previous studies have demonstrated that blood viscosity markedly decreases after smoking cessation [30]. This indicates that the elevated viscosity observed in our ever smoker population is likely a transient and modifiable physiological response to cigarette smoke rather than a permanent pathological change. Further, never smokers with COPD had the most severe baseline lung impairment (FEV_1_, FEF_25−75_%, and DLCO), but their blood viscosity remained within the normal range, and they showed no signs of secondary polycythemia such as elevated hemoglobin concentration or hematocrit. This suggests that airflow limitation alone may be insufficient to induce secondary polycythemia. A low FEV_1_ or DLCO does not necessarily indicate sustained hypoxemia that is sufficient to trigger erythrocytosis. Secondary polycythemia appears to be more closely related to persistent hypoxemia than to spirometric impairment alone. In addition, never smokers lack tobacco-derived carboxyhemoglobin, which facilitates functional tissue hypoxia by shifting the oxyhemoglobin dissociation curve to the left. Taken together, these findings suggest that smoking-related factors such as exogenous oxidative stress and carbon monoxide exposure, rather than the underlying pathophysiology of COPD, drive the increase in blood viscosity in this population.

Expanding upon our cross-sectional findings, we evaluated the longitudinal effect of blood viscosity on lung function. Our adjusted models showed that blood viscosity was not significantly associated with the 2-year changes in FEV_1_, FVC, or DLCO. This lack of association is challenging to explain directly because of the limited literature on blood viscosity and lung function. To address this gap, we interpreted our results through the lens of secondary polycythemia, which is closely linked to elevated viscosity [34]. Unfortunately, the direct longitudinal effects of polycythemia on lung-function trajectories remains poorly established. Consequently, we extrapolated data from studies examining the association between polycythemia and emphysema, which is a key structural surrogate for FEV_1_ decline [35]. However, this association remains controversial. While the SPIROMICS cohort study showed that polycythemia was associated with increased emphysema and accelerated FEV_1_ decline [36], the COPDGene cohort reported no such association in ever smokers with COPD [37]. These conflicting findings suggest that elevations in hematocrit and viscosity are recognized consequences of chronic hypoxia [38], but they may not independently drive the progression of lung tissue destruction or functional loss. Consequently, the absence of a significant association in our study is plausible and reflects the complex and potentially non-causal nature of blood viscosity in the context of chronic lung disease.

Never smokers with COPD had the highest mMRC scores and the lowest FEV_1_ and FEV_1_% predicted in our study. This pattern may reflect the distinct phenotype of non-smokers with COPD previously described in the KOCOSS cohort; non-smokers with COPD were more often female and had more comorbidities, including osteoporosis and respiratory or allergic diseases, and more tuberculosis-destroyed lungs and bronchiectasis on radiological assessment [39]. These features may contribute to dyspnea, chronic cough, sputum production, recurrent infections, reduced physical activity, and impaired quality of life independent of airflow limitation. In addition, differences in sex distribution and body size may partly explain the lower absolute FVC and FEV_1_ values of the never-smoker group; the ever-smoker COPD group was predominantly male, whereas the never-smoker COPD group included a higher proportion of females. Therefore, the greater symptom burden and functional impairment observed in never smokers with COPD in our cohort may represent this Korean non-smoking COPD phenotype, although the small size of this subgroup warrants cautious interpretation.

We also observed an unexpected positive association between male sex and improvement in FEV_1_% predicted. Although the sex distribution did not differ significantly between participants with and without complete follow-up data, this finding should be interpreted cautiously because it may reflect residual confounding or selection bias rather than a causal sex-specific effect. Other factors, including baseline characteristics, treatment patterns, smoking cessation, occupational exposure reduction, comorbidity burden, and follow-up completion, may have also contributed to this finding. Therefore, the results should be regarded as exploratory.

Our study has the following limitations. First, there was a substantial number of patients lost to follow-up. When completers were stringently defined as participants with non-missing baseline, 1-year, and 2-year FEV_1_, FVC, and DLCO data, only 102 of the 292 participants were included in the longitudinal complete-case analysis. Only four of the never smokers with COPD underwent follow-up spirometry. This high rate of loss to follow-up may have introduced selection bias. To assess potential selection bias, we compared the baseline characteristics of the participants with and without follow-up data. This analysis revealed differences in age, smoking status, and several baseline pulmonary function parameters, suggesting that attrition was not completely random. However, baseline blood viscosity, hematocrit, BMI, hs-CRP, CCI, COPD status, and four-level smoking/COPD group distribution did not differ significantly between the two groups. Therefore, selection bias and limited power may have affected the precision and generalizability of the longitudinal analysis, but the absence of a significant association between baseline blood viscosity and lung function decline is less likely to be explained solely by the differential loss to follow-up associated with blood viscosity. Second, the short-term follow-up duration for lung function tests in this study was limited to only 2 years, which may not have been long enough to observe any meaningful effects. Third, red cell distribution width was not included in the collected data. However, we measured hematocrit and assessed its correlation with blood viscosity [40]. Fourth, we performed multivariable regression analyses and extended the models to account for potential confounding factors, but residual confounding factors cannot be excluded. Detailed medication adherence data were not systematically collected, and prior exacerbation history was not incorporated into the whole-cohort model because it was not uniformly applicable to participants without COPD. Information regarding hypoxia was not available because arterial blood gas analysis was not routinely performed for this cohort. Moreover, pulmonary function testing was performed for clinically stable participants, and patients with overt clinical instability or significant hypoxemia did not generally undergo routine pulmonary function test assessment. Finally, the subgroup sizes were unequal, and the never-smoker COPD group was particularly small. This was an exploratory secondary analysis of available data from an existing prospective cohort, no formal a priori sample size calculations were performed. Accordingly, subgroup comparisons, particularly those involving never smokers with COPD, and the findings of longitudinal regression analyses should be interpreted as exploratory. This imbalance may have reduced the statistical power and limited the generalizability of our findings. Despite these limitations, our study has significant strengths, including the originality and enrollment of never smokers with COPD. As mentioned above, this is the first prospective study to explore the association of blood viscosity with the presence of COPD and longitudinal lung function decline. By including a distinct group of never smokers with COPD, we clarified the independent associations among blood viscosity, smoking status, and COPD.

In conclusion, our study demonstrated that blood viscosity is more closely associated with smoking-related hemorheological changes than with COPD. Furthermore, baseline blood viscosity had a limited value as a predictive biomarker for 2-year longitudinal lung function decline in this cohort. These findings suggest that these alterations may not be the pivotal mechanism underlying COPD progression, although smoking induces significant hemorheological changes. Further long-term studies with larger populations are warranted to fully elucidate the complex interactions between blood rheology and chronic airway disease.

## Supplementary Information

Supplementary Material 1.

## Data Availability

Upon reasonable request, the KOCOSS datasets used and/or analyzed in this study are available from the corresponding author.

## Abbreviations

BMI: body mass index
CAT: COPD Assessment Test
CCI: Charlson Comorbidity Index
COPD: chronic obstructive pulmonary disease
CRP: C-reactive protein
DLCO: diffusing capacity of the lung for carbon monoxide
EDTA: ethylenediaminetetraacetic acid
FEF25–75%: forced expiratory flow between 25% and 75% of forced vital capacity
FEV_1_: forced expiratory volume in 1 s
FEV_1_/FVC: ratio of forced expiratory volume in 1 s to forced vital capacity
FVC: forced vital capacity
hs-CRP: high-sensitivity C-reactive protein
IQR: interquartile range
IRB: Institutional Review Board
KOCOSS: Korea COPD Subgroup Study
mMRC: modified Medical Research Council
PRISm: preserved ratio impaired spirometry
RBC: red blood cell
SD: standard deviation
SGRQ-C: St. George’s Respiratory Questionnaire for COPD
SPIROMICS: SubPopulations and InteRmediate Outcome Measures In COPD Study
VIF: variance inflation factor
WBC: white blood cell
