# Supplementary material for: Blood viscosity is associated with smoking status rather than the presence of chronic obstructive pulmonary disease: findings from the Korea COPD Subgroup Study (KOCOSS)
Source: Respir Res. 2026 Aug 4;27:300. doi: 10.1186/s12931-026-03827-8 (PMC13435482; doi:10.1186/s12931-026-03827-8)
Supplement: Supplementary file 1 [file 12931_2026_3827_MOESM1_ESM.docx]

**Supplementary Table S1. Pairwise comparison for baseline characteristics of study participants**

| **Variable** | **Test** | **NS-nonCOPD vs NS-COPD** | **NS-nonCOPD vs ES-nonCOPD** | **NS-nonCOPD vs ES-COPD** | **NS-COPD vs ES-nonCOPD** | **NS-COPD vs ES-COPD** | **ES-nonCOPD vs ES-COPD** |
| --- | --- | --- | --- | --- | --- | --- | --- |
| **Sex, male** | **F** | **1.000** | **0.617** | **1.000** | **0.517** | **1.000** | **0.180** |
| **Age, years** | **W** | **0.105** | **0.519** | **0.033** | **0.044** | **0.013** | **0.016** |
| **Education** | **χ²** | **0.693** | **0.085** | **0.008** | **0.723** | **0.436** | **0.291** |
| **Pack-years** | **W** | **N/A** | **N/A** | **N/A** | **N/A** | **N/A** | **0.049** |
| **BMI, kg/m²** | **W** | **0.318** | **0.116** | **0.417** | **0.045** | **0.111** | **0.344** |
| **SBP, mmHg** | **W** | **0.503** | **0.287** | **0.784** | **0.106** | **0.408** | **0.292** |
| **DBP, mmHg** | **W** | **0.655** | **0.073** | **0.396** | **0.407** | **0.476** | **0.011** |
| **Heart rate, /min** | **W** | **0.573** | **0.057** | **0.149** | **0.583** | **0.763** | **0.745** |
| **mMRC grade (continuous)** | **W** | **0.003** | **0.842** | **0.044** | **<.001** | **0.048** | **0.011** |
| **mMRC ≥ 2 (binary)** | **F** | **1.000** | **0.508** | **0.088** | **0.347** | **1.000** | **0.008** |
| **CAT score (continuous)** | **W** | **0.073** | **0.335** | **0.033** | **0.115** | **0.574** | **0.064** |
| **CAT ≥ 10 (binary)** | **F** | **0.580** | **0.655** | **0.103** | **0.249** | **0.109** | **0.069** |
| **SGRQ-c Symptom** | **W** | **0.231** | **0.419** | **0.628** | **0.049** | **0.370** | **0.165** |
| **SGRQ-c Activity** | **W** | **0.237** | **0.863** | **0.079** | **0.181** | **0.954** | **0.022** |
| **SGRQ-c Impact** | **W** | **0.435** | **0.504** | **0.417** | **0.622** | **0.896** | **0.708** |
| **SGRQ-c Total** | **W** | **0.337** | **0.920** | **0.199** | **0.149** | **0.861** | **0.085** |
| **WBC, 10³/μL** | **W** | **0.009** | **<.001** | **0.004** | **0.950** | **0.801** | **0.969** |
| **Hemoglobin, g/dL** | **W** | **0.469** | **<.001** | **<.001** | **0.059** | **0.045** | **0.838** |
| **Hematocrit, %** | **W** | **0.778** | **<.001** | **<.001** | **0.048** | **0.045** | **0.638** |
| **Uric acid, mg/dL** | **W** | **0.151** | **<.001** | **<.001** | **0.276** | **0.110** | **0.161** |
| **AST, IU/L** | **W** | **0.867** | **0.202** | **0.126** | **0.593** | **0.496** | **0.476** |
| **ALT, IU/L** | **W** | **0.807** | **0.046** | **0.002** | **0.146** | **0.037** | **0.136** |
| **Total bilirubin, mg/dL** | **W** | **0.002** | **<.001** | **0.002** | **0.570** | **0.300** | **0.572** |
| **Creatinine, mg/dL** | **W** | **0.761** | **<.001** | **<.001** | **0.010** | **0.004** | **0.096** |
| **BUN, mg/dL** | **W** | **0.743** | **0.051** | **0.029** | **0.424** | **0.255** | **0.434** |
| **hs-CRP, mg/L** | **W** | **0.085** | **<.001** | **0.006** | **0.622** | **0.942** | **0.411** |
| **CCI score** | **W** | **0.571** | **0.054** | **0.018** | **0.664** | **0.441** | **0.368** |
| **Blood viscosity, cP** | **W** | **0.183** | **<.001** | **<.001** | **0.793** | **0.521** | **0.687** |

W = Wilcoxon rank-sum (Mann-Whitney U), F = Fisher exact, χ² = Chi-square.

N/A: Pack-years not applicable for never-smoker groups.

Bonferroni correction threshold for 6 comparisons: p < 0.008.

**Abbreviations.** BMI, body mass index; SBP, systolic blood pressure; DBP, diastolic blood pressure; mMRC, the modified Medical Research Council Dyspnea Scale; CAT score, COPD Assessment Test; SGRQ-C, COPD-specific version of St. George’s Respiratory Questionnaire; WBC, white blood count; AST, aspartate aminotransferase; ALT, alanine aminotransferase; BUN, blood urea nitrogen. NS: non-smoker; ES: ever smoker

**Supplementary Table S2. Baseline Characteristics: 2-Year Completers vs Dropouts (N=292)**

| **Variable** | **Completer (N=102)** | **Dropout (N=190)** | **p-value** |
| --- | --- | --- | --- |
| **Demographics & Clinical** |  | | |
| **Sex, male, n(%)** | 90 (88.24%) | 153 (80.53%) | 0.093 |
| **Age, years** | 65.30 ± 10.58 | 59.23 ± 14.13 | 0.001 |
| **Education** |  |  | 0.435 |
| **≤Middle school** | 28 (29.17%) | 43 (23.56%) |  |
| **High school** | 36 (37.50%) | 76 (41.76%) |  |
| **≥College** | 32 (33.33%) | 63 (34.62%) |  |
| **Pack-years** | 37.33 ± 34.04 (n=79) | 30.25 ± 22.73 (n=130) | 0.092 |
| **BMI, kg/m²** | 24.11 ± 3.69 | 24.23 ± 3.26 | 0.657 |
| **BMI category** |  |  | 0.273 |
| **<18.5** | 5 (4.90%) | 7 (3.68%) |  |
| **18.5-22.9** | 35 (34.31%) | 53 (27.89%) |  |
| **23.0-24.9** | 20 (19.61%) | 57 (30.00%) |  |
| **≥25** | 42 (41.18%) | 73 (38.42%) |  |
| **mMRC, < 2 / ≥ 2** | 88/14 | 164/23 | 0.932 |
| **CAT score** | 9.23 ± 7.44 | 8.74 ± 6.36 (n=187) | 0.942 |
| **CAT < 10 / ≥ 10** | 65/37 | 112/75 | 0.530 |
| **SGRQ-c Symptom** | 20.90 ± 18.83 (n=101) | 26.18 ± 18.99 (n=187) | 0.010 |
| **SGRQ-c Activity** | 17.54 ± 19.17 (n=101) | 17.07 ± 18.23 (n=187) | 0.987 |
| **SGRQ-c Impact** | 8.71 ± 13.90 (n=101) | 9.63 ± 12.87 (n=187) | 0.636 |
| **SGRQ-c Total** | 13.57 ± 14.08 (n=101) | 14.84 ± 13.24 (n=187) | 0.229 |
| **WBC, 10³/μL** | 6.26 ± 2.39 (n=101) | 6.43 ± 1.88 | 0.111 |
| **Hemoglobin, g/dL** | 14.18 ± 1.41 (n=101) | 14.10 ± 1.59 | 0.567 |
| **Hematocrit, %** | 42.17 ± 4.14 (n=101) | 42.05 ± 4.59 | 0.835 |
| **Uric acid, mg/dL** | 5.46 ± 1.41 (n=101) | 5.32 ± 1.42 (n=189) | 0.542 |
| **AST, IU/L** | 28.65 ± 32.44 (n=101) | 24.17 ± 9.84 | 0.133 |
| **ALT, IU/L** | 24.53 ± 18.96 (n=101) | 23.17 ± 13.55 | 0.889 |
| **Total bilirubin, mg/dL** | 0.46 ± 0.22 (n=101) | 0.47 ± 0.27 | 0.955 |
| **Creatinine, mg/dL** | 0.97 ± 0.21 (n=101) | 0.99 ± 0.52 | 0.415 |
| **BUN, mg/dL** | 17.05 ± 4.71 (n=101) | 16.11 ± 5.58 | 0.064 |
| **hs-CRP, mg/L** | 1.41 ± 2.14 (n=100) | 2.08 ± 5.35 | 0.992 |
| **CCI score** | 0.55 ± 0.98 (n=93) | 0.45 ± 0.92 (n=176) | 0.321 |
| **Blood viscosity, cP** | 9.11 ± 1.46 | 8.98 ± 1.56 | 0.696 |
| **Lung Function (median, IQR)** |  | | |
| **FVC, L** | 3.76 (3.28, 4.24) | 3.64 (3.01, 4.32) | 0.151 |
| **FVC, %pred** | 89 (82, 99) | 87 (76, 95) | 0.043 |
| **FEV1, L** | 2.61 (2.20, 2.98) | 2.59 (2.00, 3.12) | 0.660 |
| **FEV1, %pred** | 85 (77, 93) | 80.5 (67, 93) | 0.025 |
| **FEF25-75%, %pred** | 59.5 (47, 76) | 59 (39, 89) | 0.908 |
| **FEV1/FVC, %** | 69.5 (64, 74) | 72 (64, 78) | 0.045 |
| **BD FVC, L** | 3.77 (3.20, 4.31) | 3.63 (3.03, 4.27) | 0.143 |
| **BD FVC, %pred** | 89 (81, 99) | 86 (77, 96) | 0.047 |
| **BD FEV1, L** | 2.67 (2.23, 3.07) | 2.68 (2.09, 3.18) | 0.529 |
| **BD FEV1, %pred** | 88 (80, 95) | 84 (70, 94) | 0.021 |
| **BD FEF25-75%, %pred** | 65 (51, 88) | 72 (44, 95) | 0.869 |
| **BD FEV1/FVC, %** | 72 (66, 77) | 74 (66, 79) | 0.188 |
| **DLCO, mL/mmHg/min** | 16.8 (13.4, 20.7) | 17.5 (14.05, 22.6) | 0.157 |
| **DLCO, %pred** | 80.5 (67, 94) | 85 (69, 95.5) | 0.227 |
| **DLVA, mL/mmHg/min** | 3.54 (2.83, 4.08) | 3.87 (3.31, 4.45) | 0.001 |
| **DLVA, %pred** | 87 (69, 98) | 89 (78, 100) | 0.057 |
| **COPD Status & Smoking** |  | | |
| **COPD, n(%)** | 33 (32.35%) | 60 (31.58%) | 0.892 |
| **Ever-smoker, n(%)** | 86 (84.31%) | 141 (74.21%) | 0.048 |
| **Group** |  |  | 0.231 |
| **NS+COPD** | 3 (2.94%) | 13 (6.84%) |  |
| **ES+COPD** | 30 (29.41%) | 47 (24.74%) |  |
| **NS+nonCOPD** | 13 (12.75%) | 36 (18.95%) |  |
| **ES+nonCOPD** | 56 (54.90%) | 94 (49.47%) |  |

Supplementary Table S3. Pairwise comparison for baseline lung function test of study participants

| **Variable** | **Test** | **NS-nonCOPD vs NS-COPD** | **NS-nonCOPD vs ES-nonCOPD** | **NS-nonCOPD vs ES-COPD** | **NS-COPD vs ES-nonCOPD** | **NS-COPD vs ES-COPD** | **ES-nonCOPD vs ES-COPD** |
| --- | --- | --- | --- | --- | --- | --- | --- |
| **FVC, L** | **W** | **0.891** | **<.001** | **<.001** | **0.003** | **0.006** | **0.977** |
| **FVC, %pred** | **W** | **0.170** | **0.997** | **0.818** | **0.137** | **0.138** | **0.686** |
| **FEV1, L** | **W** | **0.053** | **<.001** | **0.447** | **<.001** | **0.141** | **<.001** |
| **FEV1, %pred** | **W** | **0.003** | **0.826** | **<.001** | **<.001** | **0.267** | **<.001** |
| **FEF25-75%, %pred** | **W** | **<.001** | **0.367** | **<.001** | **<.001** | **0.518** | **<.001** |
| **FEV1/FVC** | **W** | **<.001** | **0.001** | **<.001** | **<.001** | **0.795** | **<.001** |
| **BD FVC, L** | **W** | **0.869** | **<.001** | **<.001** | **0.009** | **0.007** | **0.432** |
| **BD FVC, %pred** | **W** | **0.150** | **0.769** | **0.484** | **0.158** | **0.090** | **0.218** |
| **BD FEV1, L** | **W** | **0.097** | **<.001** | **0.517** | **0.002** | **0.194** | **<.001** |
| **BD FEV1, %pred** | **W** | **0.004** | **0.741** | **<.001** | **0.002** | **0.172** | **<.001** |
| **BD FEF25-75%, %pred** | **W** | **0.001** | **0.250** | **<.001** | **<.001** | **0.500** | **<.001** |
| **BD FEV1/FVC** | **W** | **0.002** | **0.003** | **<.001** | **0.002** | **0.991** | **<.001** |
| **DLCO, mL/mmHg/min** | **W** | **0.539** | **0.037** | **0.711** | **0.103** | **0.716** | **0.011** |
| **DLCO, %pred** | **W** | **0.042** | **0.073** | **<.001** | **0.261** | **0.973** | **0.006** |
| **DLVA, mL/mmHg/min** | **W** | **0.585** | **<.001** | **<.001** | **0.414** | **0.041** | **0.004** |
| **DLVA, %pred** | **W** | **0.457** | **0.061** | **0.357** | **0.972** | **0.262** | **0.012** |

W = Wilcoxon rank-sum (Mann-Whitney U), F = Fisher exact, χ² = Chi-square.

N/A: Pack-years not applicable for never-smoker groups.

Bonferroni correction threshold for 6 comparisons: p < 0.008.

**Abbreviations** FVC, Forced Vital Capacity; % pred, percent predicted; FEV1, Forced Expiratory Volume in 1 Second; FEF25-75%, Forced Expiratory Flow; FEV1/FVC, Ratio of Forced Expiratory Volume in 1 Second to Forced Vital Capacity; BD, Bronchodilator; DLCO, Diffusing Capacity of the Lung for Carbon Monoxide; DLVA, Alveolar Volume Adjusted DLCO. NS: non-smoker; ES: ever smoker

**Supplementary Table S4. Correlation analyses of hematocrit, blood viscosity, and pack-years.**

| **Analysis** | **Subgroup** | **N** | **Method** | **Coefficient** | **p-value** |
| --- | --- | --- | --- | --- | --- |
| **Hct vs Blood viscosity** | **Overall** | 291 | Pearson r | 0.64 | <0.001 |
|  | **Never-smoker** | 65 | Pearson r | 0.702 | <0.001 |
|  | **Ever-smoker** | 226 | Pearson r | 0.596 | <0.001 |
|  | **Current smoker** | 86 | Pearson r | 0.455 | <0.001 |
|  | **Ex-smoker** | 140 | Pearson r | 0.668 | <0.001 |
| **Pack-years vs Blood viscosity** | **Ever-smoker, all** | 209 | Spearman ρ | −0.091 | 0.189 |
|  | **Ever-smoker with COPD** | 60 | Spearman ρ | 0.029 | 0.825 |
|  | **Ever-smoker without COPD** | 149 | Spearman ρ | −0.136 | 0.099 |

N varies across analyses because of missing values in hematocrit or pack-year data. Pack-year analyses were restricted to ever-smokers with available pack-year information. Pearson correlation was used for hematocrit and blood viscosity because both variables were approximately continuous, whereas Spearman correlation was used for pack-years because of its skewed distribution.

**Supplementary Table S5. Extended regression models for 2-year changes in FEV1, FVC, and DLCO % predicted**

| **FEV1 % Predicted 2-Year Change: Extended Regression Models** | | | | | | | |
| --- | --- | --- | --- | --- | --- | --- | --- |
| **Variable** | **M1 (Original)** | **M2 (+BMI, Any inhaler)** | **M3 (M2+CRP)** | **M4 (M2+WBC)** | **M5 (+BMI, LABA/LAMA)** | **M6 (M5+CRP)** | **M7 (M5+WBC)** |
| Age | -0.08 (-0.25, 0.09) | -0.09 (-0.25, 0.07) | -0.08 (-0.24, 0.09) | -0.09 (-0.25, 0.08) | -0.08 (-0.24, 0.09) | -0.07 (-0.24, 0.10) | -0.08 (-0.25, 0.09) |
| Male sex | 7.58 (1.70, 13.47) | 9.80 (3.77, 15.83) | 9.53 (3.45, 15.60) | 9.86 (3.63, 16.09) | 8.96 (2.93, 14.99) | 8.71 (2.67, 14.74) | 9.08 (2.82, 15.35) |
| Never Smoker+COPD vs ref | 11.05(-0.80, 22.90) | 13.04 (1.35, 24.73) | 12.83 (1.11, 24.54) | 13.02 (1.26, 24.79) | 11.84(-0.00, 23.69) | 11.79(-0.05, 23.63) | 11.84(-0.09, 23.76) |
| Ever Smoker+COPD vs ref | -0.30 (-6.02, 5.41) | -1.52 (-7.24, 4.19) | -0.89 (-6.68, 4.91) | -1.58 (-7.52, 4.36) | -1.07 (-6.87, 4.74) | -0.47 (-6.34, 5.39) | -1.19 (-7.26, 4.87) |
| Ever Smoker+nonCOPD vs ref | -0.09 (-5.46, 5.28) | -1.06 (-6.36, 4.25) | -0.53 (-5.91, 4.85) | -1.08 (-6.45, 4.29) | -0.75 (-6.12, 4.62) | -0.26 (-5.69, 5.17) | -0.80 (-6.24, 4.64) |
| Blood viscosity | 0.34 (-0.80, 1.48) | 0.58 (-0.57, 1.74) | 0.87 (-0.37, 2.11) | 0.57 (-0.61, 1.76) | 0.61 (-0.56, 1.78) | 0.93 (-0.32, 2.18) | 0.59 (-0.61, 1.80) |
| BMI |  | -0.43 (-0.94, 0.07) | -0.45 (-0.95, 0.06) | -0.44 (-0.96, 0.08) | -0.44 (-0.95, 0.07) | -0.45 (-0.97, 0.06) | -0.45 (-0.97, 0.08) |
| Medication |  | -4.42 (-9.15, 0.31) | -3.94 (-8.90, 1.02) | -4.39 (-9.20, 0.42) | -1.92 (-5.65, 1.81) | -1.42 (-5.23, 2.39) | -1.86 (-5.68, 1.96) |
| CRP |  |  | -0.53 (-1.33, 0.27) |  |  | -0.56 (-1.37, 0.25) |  |
| WBC |  |  |  | 0.03 (-0.71, 0.77) |  |  | 0.06 (-0.70, 0.82) |
| Viscosity p-value | 0.557 | 0.319 | 0.165 | 0.337 | 0.303 | 0.143 | 0.329 |
| R² | 0.127 | 0.189 | 0.203 | 0.189 | 0.165 | 0.183 | 0.165 |

| **FVC % Predicted 2-Year Change: Extended Regression Models** | | | | | | | |
| --- | --- | --- | --- | --- | --- | --- | --- |
| **Variable** | **M1 (Original)** | **M2 (+BMI, Any inhaler)** | **M3 (M2+CRP)** | **M4 (M2+WBC)** | **M5 (+BMI, LABA/LAMA)** | **M6 (M5+CRP)** | **M7 (M5+WBC)** |
| Age | -0.13 (-0.27, 0.02) | -0.13 (-0.28, 0.01) | -0.12 (-0.27, 0.03) | -0.14 (-0.28, 0.01) | -0.13 (-0.28, 0.02) | -0.12 (-0.27, 0.03) | -0.13 (-0.28, 0.01) |
| Male sex | 4.08 (-1.10, 9.24) | 5.11 (-0.33, 10.54) | 4.86 (-0.60, 10.32) | 4.57 (-1.04, 10.19) | 4.49 (-0.91, 9.89) | 4.24 (-1.16, 9.64) | 4.04 (-1.51, 9.60) |
| Never Smoker+COPD vs ref | 15.47(5.06, 25.88) | 16.39(5.66, 27.12) | 16.18(5.43, 26.94) | 16.52(5.73, 27.30) | 15.92(5.12, 26.71) | 15.87(5.09, 26.65) | 15.94(5.10, 26.79) |
| Ever Smoker+COPD vs ref | -0.11 (-5.13, 4.91) | -1.06 (-6.28, 4.17) | -0.47 (-5.81, 4.86) | -1.48 (-6.83, 3.88) | -0.42 (-5.74, 4.90) | 0.18 (-5.22, 5.58) | -0.70 (-6.16, 4.76) |
| Ever Smoker+nonCOPD vs ref | 1.09 (-3.63, 5.80) | 0.35 (-4.52, 5.23) | 0.68 (-4.31, 5.66) | 0.13 (-4.84, 5.11) | 0.95 (-3.86, 5.76) | 1.31 (-3.70, 6.33) | 0.79 (-4.12, 5.70) |
| Blood viscosity | 0.20 (-0.80, 1.20) | 0.28 (-0.76, 1.33) | 0.55 (-0.56, 1.66) | 0.37 (-0.69, 1.43) | 0.29 (-0.76, 1.35) | 0.60 (-0.52, 1.72) | 0.36 (-0.71, 1.43) |
| BMI |  | -0.16 (-0.61, 0.30) | -0.17 (-0.63, 0.28) | -0.11 (-0.58, 0.35) | -0.14 (-0.60, 0.32) | -0.16 (-0.61, 0.30) | -0.11 (-0.58, 0.36) |
| Medication |  | -2.71 (-6.97, 1.55) | -2.27 (-6.74, 2.19) | -2.98 (-7.29, 1.33) | 0.19 (-3.15, 3.53) | 0.67 (-2.73, 4.07) | -0.02 (-3.43, 3.40) |
| CRP |  |  | -0.49 (-1.21, 0.23) |  |  | -0.54 (-1.27, 0.19) |  |
| WBC |  |  |  | -0.28 (-0.95, 0.38) |  |  | -0.22 (-0.90, 0.46) |
| Viscosity p-value | 0.686 | 0.587 | 0.329 | 0.492 | 0.579 | 0.288 | 0.507 |
| R² | 0.144 | 0.165 | 0.180 | 0.172 | 0.148 | 0.171 | 0.152 |

| **DLCO % Predicted 2-Year Change: Extended Regression Models** | | | | | | | |
| --- | --- | --- | --- | --- | --- | --- | --- |
| **Variable** | **M1 (Original)** | **M2 (+BMI, Any inhaler)** | **M3 (M2+CRP)** | **M4 (M2+WBC)** | **M5 (+BMI, LABA/LAMA)** | **M6 (M5+CRP)** | **M7 (M5+WBC)** |
| Age | -0.26 (-0.55, 0.02) | -0.27(-0.55, 0.02) | -0.24(-0.53, 0.05) | -0.28 (-0.57, 0.01) | -0.27 (-0.56, 0.01) | -0.25 (-0.53, 0.04) | -0.28 (-0.57, 0.01) |
| Male sex | 4.65 (-5.34, 14.64) | 5.44 (-5.14, 16.01) | 4.54 (-5.96, 15.05) | 4.32 (-6.46, 15.10) | 4.43 (-6.08, 14.95) | 3.74 (-5.58, 13.06) | 3.55 (-7.28, 14.38) |
| Never Smoker+COPD vs ref | 5.26(-14.85, 25.38) | 5.90(-14.48, 26.29) | 5.24(-15.08, 25.56) | 6.18(-14.33, 26.70) | 5.61(-14.79, 26.02) | 5.49(-14.70, 25.67) | 5.66(-14.91, 26.23) |
| Ever Smoker+COPD vs ref | -0.07 (-8.80, 8.66) | -0.77 (-9.74, 8.21) | 0.34 (-8.89, 9.57) | -1.47(-10.75, 7.81) | -0.45 (-9.51, 8.62) | 1.07 (-8.17, 10.31) | -0.79 (-10.12, 8.54) |
| Ever Smoker+nonCOPD vs ref | -0.07 (-7.40, 7.27) | -0.83 (-8.35, 6.70) | 0.07 (-7.67, 7.80) | -1.33 (-9.10, 6.45) | -0.35 (-8.04, 7.34) | 0.72 (-7.17, 8.60) | -0.64 (-8.52, 7.25) |
| Blood viscosity | -0.07 (-2.00, 1.87) | -0.09 (-2.12, 1.93) | 0.47 (-1.68, 2.62) | 0.08 (-1.98, 2.14) | -0.09 (-2.12, 1.94) | 0.55 (-1.59, 2.69) | 0.04 (-2.04, 2.11) |
| BMI |  | 0.00 (-0.88, 0.88) | -0.03 (-0.91, 0.85) | 0.09 (-0.81, 1.00) | 0.05 (-0.84, 0.93) | 0.02 (-0.86, 0.90) | 0.11 (-0.80, 1.02) |
| Medication |  | -3.74 (-12.02, 4.55) | -1.88 (-10.52, 6.77) | -4.29 (-12.67, 4.10) | 2.08 (-4.37, 8.53) | 3.42 (-3.09, 9.94) | 1.67 (-4.93, 8.26) |
| CRP |  |  | -0.85 (-2.25, 0.55) |  |  | -0.97 (-2.37, 0.42) |  |
| WBC |  |  |  | -0.59 (-1.89, 0.71) |  |  | -0.43 (-1.74, 0.88) |
| Viscosity p-value | 0.947 | 0.929 | 0.665 | 0.939 | 0.930 | 0.610 | 0.970 |
| R² | 0.054 | 0.064 | 0.076 | 0.073 | 0.059 | 0.087 | 0.064 |

ref = Never-smoker without COPD (group 3). Values: beta (95% CI).

M1: age+sex+group+viscosity; M2: M1+BMI+any_inhaler; M3: M2+CRP; M4: M2+WBC

M5: M1+BMI+LABA/LAMA; M6: M5+CRP; M7: M5+WBC. PROC GLM, group (ref="3").

**Supplementary Table S6. Variance inflation factors for the primary Table 3 regression model**

| **Variable** | **VIF** |
| --- | --- |
| Age | 1.09 |
| Sex, male | 1.40 |
| Never-smoker + COPD | 1.24 |
| Ever-smoker + COPD | 2.64 |
| Ever-smoker + non-COPD | 2.77 |
| Blood viscosity | 1.13 |
| **Maximum VIF** | **2.77** |

Model: age + sex + group(ref=NS+nonCOPD) + blood viscosity
